# Communally Nesting Migratory Birds Create Ecological Hot-Spots in Tropical Australia

**DOI:** 10.1371/journal.pone.0162651

**Published:** 2016-10-05

**Authors:** Daniel J. D. Natusch, Jessica A. Lyons, Gregory Brown, Richard Shine

**Affiliations:** 1 School of Life and Environmental Sciences, University of Sydney, Sydney, New South Wales, Australia; 2 Resource Evaluation and Development, Bamaga, Queensland, Australia; University of Sydney, AUSTRALIA

## Abstract

Large numbers of metallic starlings *(Aplonis metallica*) migrate annually from New Guinea to the rainforests of tropical Australia, where they nest communally in single emergent trees (up to 1,000 birds). These aggregations create dense and species-rich faunal “hot-spots”, attracting a diverse assemblage of local consumers that utilise this seasonal resource. The starlings nested primarily in poison-dart trees (*Antiaris toxicaria*) near the rainforest-woodland boundary. Surveys underneath these colonies revealed that bird-derived nutrients massively increased densities of soil invertebrates and mammals (primarily wild pigs) beneath trees, year-round. Flying invertebrates, nocturnal birds, reptiles, and amphibians congregated beneath the trees when starlings were nesting (the wet-season). Diurnal birds (primarily cockatoos and bush turkeys) aggregated beneath the trees during the dry-season to utilise residual nutrients when the starlings were not nesting. The abundance of several taxa was considerably higher (to > 1000-fold) under colony trees than under nearby trees. The system strikingly resembles utilisation of bird nesting colonies by predators in other parts of the world but this spectacular system has never been described, emphasizing the continuing need for detailed natural-history studies in tropical Australia.

## Introduction

In most ecosystems, the spatial and temporal distribution of resources drives faunal distributions [1, 2]. All animals live in spatially and temporally heterogeneous landscapes, varying greatly in productivity, resource abundance and species density. Such heterogeneity is often subtle within habitats, but sometimes, concentrations of specific resources create dramatic impacts such that small sites become the focus for a high proportion of all faunal activity [3]. Typical examples include aggregations around water bodies in arid landscapes or at nutrient-rich patches in nutrient-poor landscapes [4, 5]. Some faunal concentrations are also seasonal, such as Nile crocodiles (*Crocodylus niloticus*) gathering at points in rivers where wildebeest cross, or grizzly bears (*Ursus arctos* ssp.) congregating at salmon spawning sites [6, 7]. These resource concentrations can influence vital population parameters such as rates of reproduction and survival [6, 8]. Massive aggregations of wildlife also are significant for conservation (e.g. vulnerable to threatening processes), management (e.g., over-exploitation by hunters) and tourism (e.g., support major tourism initiatives; [3, 8, 7, 9]).

Despite their spectacular nature and high public profile, these systems sometimes are poorly studied. We describe aggregations of wildlife that gather to exploit seasonally available resources (nesting colonies of metallic starlings, *Aplonis metallica*). As in communally-nesting African sociable weavers (*Philetairus socius*) and Peruvian yellow-rumped caciques (*Cacicus cela*), the high biomass of birds attracts diverse predators [10, 11]. Understanding of ecological factors affecting colonies are well known [11, 12]—but indirect impacts of colonies on other species are poorly known, except for anecdotal accounts [13, 14]. We compared the assemblages of animals using colony sites, compared to nearby areas, within rainforests in tropical Australia.

## Materials and Methods

### Ethics statement

The University of Sydney animal ethics committee approved this research (approval number: L04/3-2013/3/5969).

### Metallic starlings

**Fig 1:**
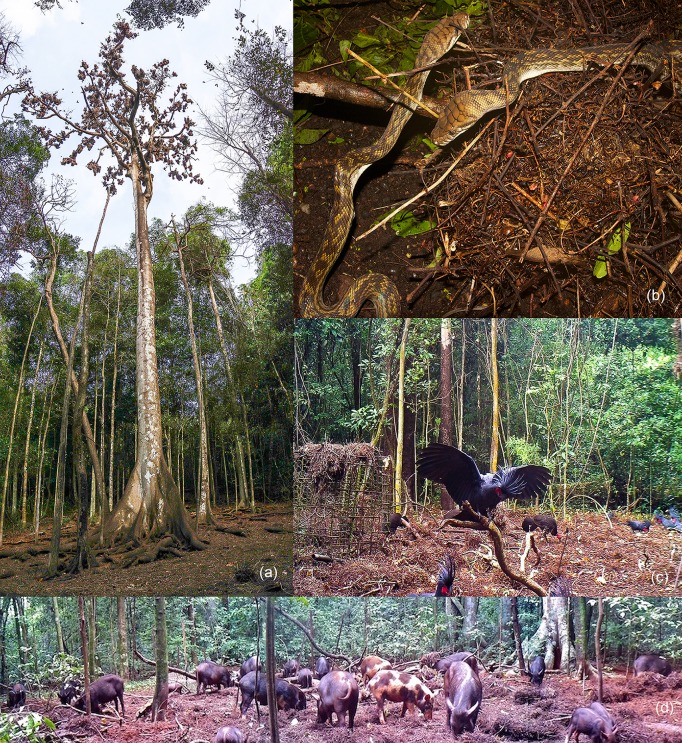
Communal nesting colonies of *Aplonis metallica* (metallic starlings) in emergent rainforest trees attract other wildlife. (a) A colony tree from ground level; (b) two *Morelia amethistina* (scrub pythons) feeding on *A*. *metallica* chicks at a fallen nest at night; (c) birds *Alectura lathami* (brush turkeys); *Probosciger aterrimus* (palm cockatoos) under a colony tree by day; and (d) *Sus scrofa* (pigs) under a colony tree by day.

Metallic starlings (hereafter referred to as “starlings”) are small glossy-black birds (22 cm; 61 g), from northeastern Australia (Queensland), New Guinea, the Moluccas, and Solomon Islands [15]. These birds closely associate with rainforests, feeding predominately on the fruits and seed arils of rainforest trees [16]. Between November and April starlings nest in large colonies (>1,000 individuals) within single emergent trees where they raise young within suspended dome-shaped nests made of bark, grasses and small vines [15]. Starlings nest in the same trees each season (one colony tree remained active for > 15 years; Natusch unpubl. data). A carpet of fallen seeds and bird guano creates an area of open ground (~ 10 x 14 m) directly beneath each colony, where it is common to find fallen eggs and chicks during the breeding season (Fig 1). After the last fledglings leave the nests, the starlings migrate to New Guinea until they return the following season [13, 15].

### Study area

**Fig 2:**
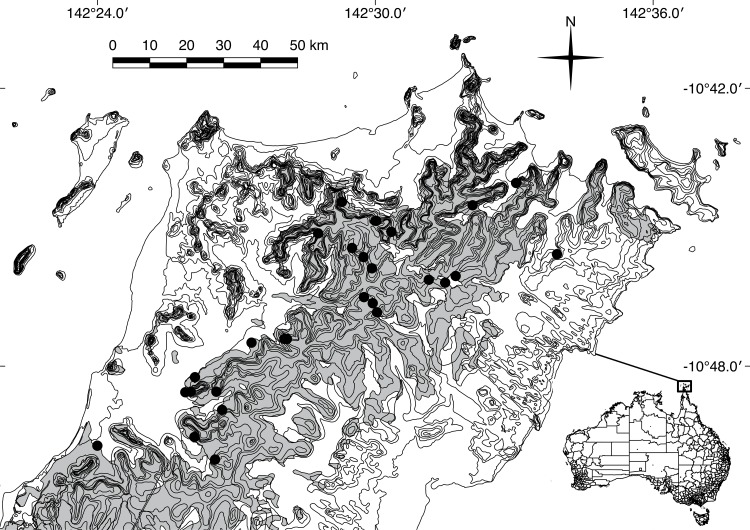
Map of the Lockerbie Scrub. Showing the boundary between closed rainforest (grey) and open woodlands (white), and location of *Aplonis metallica* (metallic starling) colonies (black circles).

The Lockerbie Scrub is a 130 km^2^ area of semi-deciduous notophyll vine forest, at the extreme northern tip of Cape York Peninsula in Queensland, Australia (Fig 2). The vegetation is comprised predominately of closed rainforest, interspersed with and surrounded by open woodlands dominated by *Corymbia tessellaris*, *C*. *clarksoniana* and/or *Eucalyptus brassiana* [17]. The climate is highly seasonal, with a mean annual rainfall of 1744 mm (range = 1268 to 3184 mm), largely falling during the summer monsoon (December to April) while the rest of year remains hot and dry with frequent fires [18].

### Seasonal timing

Based on five years of observations at our field site (2007, 2008, 2013, 2014, 2015), starlings begin nesting around mid-November. They remain at the trees for the summer monsoon, during which time a pair of starlings raises up to three broods before leaving at the beginning of April (based on the presence of eggshells and chicks beneath the colony at three separate intervals throughout the nesting season). We defined the “nesting season” as 11 November to 1 April (mean timing of nesting at our study site), and the remainder of the year as the “off-season”. We collected data between November 2013 and April 2015, which consisted of two nesting seasons and the intervening off-season when no starlings were present at the colony trees.

### Spatial distribution of colony trees

We initially located colony trees from ground level using sight and bird sound (a colony can be heard up to 300 m away), but also systematically surveyed the Lockerbie Scrub from a helicopter. If a colony tree was located, its location was recorded on a hand-held GPS unit (Garmin 60SCx). We then walked to each tree to confirm whether or not a colony was present, and estimated tree heights, identified species, and estimated number of nests in the colony. We were confident that we located most of the starling nesting colonies in the Lockerbie Scrub during our surveys.

We overlaid colony tree locations onto Regional Ecosystem maps for Queensland in QGIS (version 1.8.0) to determine the broad vegetation types at each colony tree [17, 19]). To establish whether starlings associated with colony trees near the rainforest edge, we used QGIS to generate 100 random points within the rainforest regional ecosystem and measured the distance of each point to the rainforest edge. We compared these to the actual distances of each colony tree to the rainforest edge.

### Survey methods

We used four methods to quantify the abundance and diversity of fauna present at starling colonies throughout the year. To test whether faunal concentrations beneath colony trees were related to the presence of the bird colony, we also surveyed an identical number of nearby trees that were not used by starlings. For each colony tree, we selected a “random” tree 100–200 m away, and similar to the colony tree in height, girth and, if possible, species. Our surveys recorded animals in the “dead zone” (an area of little or no vegetation and with large amounts of seed and guano dropped by the starlings above; Fig 1) beneath each colony tree, with a mean area of 10 x 14 m (range: 4 x 8 m to 21 x 22 m). We recorded animals under “random” trees in a similar 10 x 14 m area, directly beneath the chosen tree. Where possible we chose random trees with an open understory, to resemble the “dead zone” underneath the colony trees. Although on a few occasions the rainforest vegetation did not allow a correspondingly large field of view underneath random trees (which may have influenced our ability to detect animals), this bias probably had little influence our results. To record faunal concentrations we used the following methods.

#### Abundance of invertebrates

We randomly selected four colony trees and four random trees and surveyed them for flying and soil-dwelling invertebrates. To record flying invertebrates we stapled an 80 x 4 cm length of Sanli^®^ fly paper to a tree branch 1.5 m above the ground. We allowed the fly paper to hang freely from the branch for 24 hours, and counted all invertebrates adhered to it. We repeated this procedure twice during the nesting-season and twice during the off-season. To record soil invertebrates, we collected 50 g of soil from two locations beneath each colony tree and each random tree (total of 100 g of soil from each tree). We placed the soil in a Tullgren funnel beneath a heat-lamp overnight, and counted invertebrates the next morning. We then placed the soil in a glass dish and visually located and counted remaining invertebrates.

#### Abundance of diurnal fauna

For one-year (January to December 2014) we surveyed six of the logistically most accessible colony trees in our study area, and their corresponding random trees, using Moultrie Panoramic 150™ infrared trail cameras. We deployed cameras with a 150° field of view on tree trunks (40 cm above ground) to provide an unobstructed view of either the colony tree or the random tree. This method recorded all animals within the 10 x 14 m clear area beneath the colony trees, as well as a corresponding area under random trees. To survey multiple trees each month, we deployed cameras at a tree for 7 to 10 days. After this period, we removed the cameras, changed their batteries, and deployed them at another site. This way most trees were surveyed every month. Cameras were set to motion detection, with a minimum of 5 minutes between successive shots (to minimize multiple photographs of the same individuals). Many animals may have spent longer than five minutes under the starling colony, so we included the number of photos taken per day as a covariate in our analyses.

#### Abundance of nocturnal fauna

Between November 2013 and April 2015 we surveyed a subset of colony trees and nearby random trees for nocturnal animals. In the first survey year we visited 12 colonies (and their associated random trees) one to five times per week. During our second survey year, we regularly visited only eight colony trees and their associated random trees. We conducted surveys on foot (1930–2230 h) and searched the 10 x 14 m area of ground (and the immediate surrounding vegetation) underneath each tree for 1–3 minutes with the aid of a head-torch. We conducted surveys during the nesting-season and the off-season (but more often in the nesting season, when nocturnal predators were most abundant).

#### Statistical analyses

We used analysis of variance (ANOVA) to determine whether colony trees were distributed closer to rainforest edges than would be expected by chance. We square-root transformed the distance of colony tree locations and randomly generated point distances from the rainforest edge to meet assumptions of normality and homogeneity of variance. Because our faunal counts of all taxa contained a high proportion of zeros (many surveys recorded no animals, while others recorded hundreds), we analyzed count data using generalized linear models with negative binomial distributions and log-link functions [20]. To control for multiple counts taken at each tree, we incorporated tree ID into models as a random effect. To examine differences in faunal counts between seasons (nesting-season vs. off-season) and tree types (colonies vs. random trees), these factors, and the interaction between them, were also included in the models. Separate analyses were carried out on counts for each of the four groups of taxa (mammals, birds, reptiles and amphibians). Faunal count data were analyzed using the GLIMMIX procedure in SAS 9.4 (SAS Institute Cary NC). All other analyses were conducted using JMP Pro 11.0 (SAS Institute Cary NC).

## Results

### Spatial distribution and attributes of colony trees

Of 27 starling colonies located in the Lockerbie Scrub, all but one were within rainforest. The exception was in woodland on the rainforest edge. Starling colonies were closer to the rainforest edge than expected (*F*_1,126_ = 6.63, *P* = 0.01; Fig 2). Starlings nested in only three tree species: poison-dart trees (*Antiaris toxicaria* 71%); milky pines (*Alstonia scholaris* 19%); and Morton Bay ash (*Corymbia tesselaris* 10%). Colony trees were always tall (mean height 25 m; taller than most other trees in the study area; Natusch et al. under review), with smooth bark, large trunks and branches separated from the surrounding canopy (Fig 1). The mean number of starling nests per tree was 399 (standard deviation: 223; range: 33–815).

### Abundance of invertebrates

**Fig 3:**
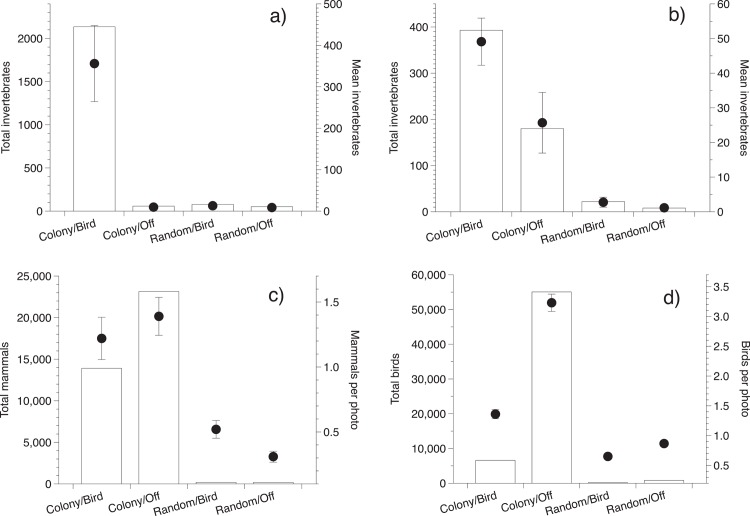
Faunal abundance under *Aplonis metallica* (metallic starling) colony and random trees during the bird nesting and off seasons. (a) Invertebrates in sticky-trap samples and (b) soil; camera-trap counts of (c) mammals and (d) birds as a function of location (colony trees vs. random trees) and season (bird nesting season vs. off-season). Figures for each taxon show total number of individuals recorded (columns) and mean (±SE) values (points) of animals per survey.

Soil samples from colony and random trees contained abundant isopterans, coleopterans and formicidans, while dipterans constituted most flying invertebrates captured by the flytraps. A significant interaction between tree type and season confirmed that flying invertebrates were more abundant under colony trees than random trees, but only during the nesting season (*F*_1,18_ = 40.0, *P* < 0.0001; Fig 3A). Soil invertebrates were most abundant beneath colony trees (*F*_1,22_ = 70.1, *P* < 0.0001) and during the nesting season (*F*_1,18_ = 4.54, *P* = 0.045; interaction NS; Fig 3B).

### Abundance of diurnal predators

Over 880 camera-trap days (20,053 photographs) we identified more than 95,000 animals representing 23 species (Table 1). Most (95%) photographs were taken under colony trees rather than random trees, because most (99%) animals were recorded beneath colony trees. The maximum number of individual animals in a single photograph was 50 under a colony tree versus nine under a random tree. Brush turkeys (*Alectura lathami*; 55%) and pigs (*Sus scrofa*; 38%) comprised most (93%) animals recorded under trees (Table 1). Cameras detected only small numbers of reptiles and amphibians, and always when an endothermic animal was also present (presumably, triggering the camera)(Table 1). For this reason, and because most reptiles and amphibians were recorded during our nocturnal surveys, we omitted them from analyses. After correcting for number of photos, we found significantly more mammals at colony trees than at random trees, a difference greater during the off season than the nesting season (interaction term: *F*_1,898_ = 7.2, *P* = 0.007; Fig 3C). Like mammals, more birds were recorded at colony trees than random trees (*F*_1,898_ = 72.8, *P* < 0.0001), especially during the off season (*F*_1,898_ = 48.5, *P* < 0.0001; Fig 3D). The presence of mammals was negatively associated with the presence of other birds (i.e., non-starlings)(*r*^2^ = 0.04; *P* < 0.0001).

**Table 1:** Species recorded underneath *Aplonis metallica* (metallic starling) colonies and nearby random trees using camera traps and nocturnal surveys.

Species	Camera traps		Nocturnal surveys	
	Colony	Random	Colony	Random
**Mammals**				
Pig, *Sus scrofa*	36,075	235	101	0
Dingo, *Canis lupis dingo*	244	34	0	0
Cattle, *Bos taurus*	99	12	0	0
Agile wallaby, *Macropus agilis*	49	46	0	0
Cape York Melomy, *Melomys capensis*	15	4	20	2
Southern Brown Bandicoot, *Isoodon obesulus peninsulae*	31	16	1	0
Cape York Rat, *Rattus leucopus*	15	0	6	0
Short-beaked Echidna, *Tachyglossus aculeatus*	9	9	3	1
White tailed rat, *Uromys caudimaculatus*	6	0	3	0
Horse, *Equus ferus caballus*	8	5	0	0
Wild dog, *Canis lupis*	5	0	0	0
Spiny haired bandicoot, *Echymipera rufescens*	0	0	2	0
Striped possum, *Dactylopsila trivirgata*	0	0	1	0
**Birds**				
Brush turkey, *Alectura lathami*	52,609	962	0	0
Palm cockatoo, *Probosciger aterrimus*	2232	2	0	0
Sulpher-crested cockatoo, *Cacatua galerita*	2009	1	140	0
Emerald dove, *Chalcophaps indica*	1094	0	0	0
Bar shouldered dove, *Geopelia humeralis*	293	0	0	0
Orange-footed Scrub Fowl, *Megapodius reinwardt*	264	50	0	0
Noisy pitta, *Pitta versicolor*	3	0	0	0
Red Goshawk, *Erythrotriorchis radiatus*	3	0	0	0
Buff-breasted paradise Kingfisher, *Tanysiptera sylvia*	3	0	0	0
Rufuous Owl, *Ninox rufa*	0	0	22	0
Grey Goshawk, *Accipiter novaehollandiae*	2	0	0	0
Blue winged Kookaburra, *Dacelo leachii*	1	0	0	0
**Reptiles**				
Brown tree snake, *Boiga irregularis*	0	0	638	1
Amethystine Python, *Morelia amethistina*	0	0	374	1
Slaty Grey Snake, *Stegonotus cucullatus*	0	0	224	4
Water Python, *Liasis fuscus*	0	0	18	0
Tree Dtella, *Gehyra dubia*	0	0	10	0
Giant tree gecko, *Psuedothecadactylus australis*	0	0	8	0
Brown headed snake, *Furina tristis*	0	0	8	1
Blue-tailed monitor, *Varanus doreanus*	1	1	1	0
Black-headed python, *Aspidites melanocephalus*	0	0	1	0
Spotted python, *Antaresia maculosa*	0	0	1	0
Carpet python, *Morelia spilota*	0	0	1	0
Blind snake, *Anilios polygrammicus*	0	0	1	0
**Amphibians**				
Cane toads, *Rhinella marina*	0	0	4165	3
White-lipped tree frog, *Litoria infrafrenata*	0	0	69	2
Northern Banjo Frog, *Limnodynastes terraereginae*	0	0	11	1
Marbled Frog, *Limnodynastes convexiusculus*	0	0	7	0
Green tree frog, *Litora caerulea*	0	0	5	0
**Total**	**95,070**	**1377**	**5,841**	**16**

### Abundance of nocturnal predators

**Fig 4:**
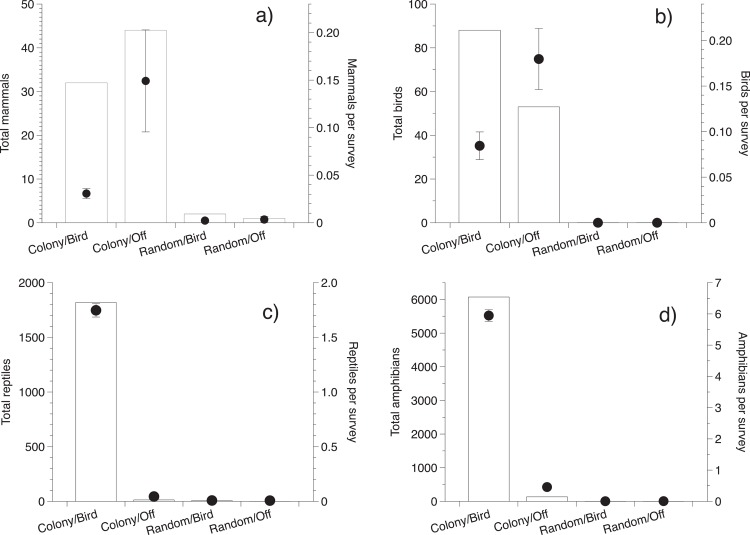
**Abundance of (a) nocturnal mammals (b), birds (c) reptiles, and (d) amphibians at colony and random trees during the bird nesting season and off-season.** Figures for each taxon show total number of individuals recorded (columns) and mean (±SE) values (points) of animals per survey.

During 1,982 nocturnal surveys of colony and random trees, we recorded 5,819 animals of 25 species (8 mammals, 2 birds, 10 reptiles and 5 amphibians; Table 1). Cane toads (*Rhinella marina*) (73%) and snakes of several species (22%) comprised most nocturnal survey records. Mammals were primarily pigs (N = 101) and Cape York melomys (*Melomys capensis*; N = 20) but with small numbers of other species (Table 1). Mammals were more common under colony trees than under random trees (*F*_1,2504_ = 24.1, *P* < 0.0001) and were more common during the off-season than the nesting season (*F*_1,2504_ = 32.6, *P* < 0.0001; interaction term NS, *P* = 0.74; Fig 4A). Of 162 records of birds surveyed at night under colony trees, 140 were sulpher-crested cockatoos (*Cacatua galerita*) and 22 were rufous owls (*Ninox rufa*). Birds were more common in the off-season than the nesting season (*F*_1,2504_ = 16.6, *P* < 0.0001). Although total counts were lower in the nesting season, presence was higher (interaction NS, *P* = 0.98; Fig 4B). We did not record any birds at random trees during our surveys (Table 1). There was a significant interaction between tree type and season, related to reptile counts (*F*_1,2502_ = 20.0, *P* < 0.0001), with high counts only at the colony trees and only during the nesting season (Fig 4C). Records of anurans under colony trees were dominated by cane toads (*R*. *marina*; N = 4,165) and white-lipped tree frogs (*Litoria infrafrenata*; N = 69). Like reptiles, anurans were far more abundant beneath colony trees than random trees, especially during the nesting season (interaction term: *F*_1,2482_ = 13.5, *P* = 0.0002; Fig 4D).

### Influence of colony size on animal abundance

The number of diurnal mammals beneath colony trees was not affected by the number of starling nests in the tree (*F*_1,555_ = 1.9, *P* = 0.17). The same was not true for birds, which were recorded in greater numbers beneath trees with more nests, and this effect was more pronounced during the nesting season than the off season (interaction term: *F*_1,555_ = 13.5, *P* = 0.0003). Of the nocturnal animals, only the number of reptiles observed at trees in the nesting season was positively correlated with the number of nests in the colony (*F*_1,982_ = 47.2, *P* < 0.0001). All other p-values > 0.05.

## Discussion

Each year, breeding colonies of metallic starlings alter the spatial and temporal heterogeneity of wildlife distributions in the rainforests of Cape York. Our surveys documented considerable abundances of soil invertebrates, flying insects, amphibians, reptiles, birds and mammals under bird-nesting trees. There were no similar aggregations under otherwise-similar trees that lacked bird colonies. Moreover, many predators aggregated at colony sites only or primarily at times of year when the birds were nesting. The magnitude of the effect was extraordinary, with densities of many taxa 100 to 1000-fold higher under colony trees than elsewhere.

Starling colonies are important for a diverse range of wildlife taxa, which utilize the colonies differently. Those differences affect seasonal patterns of abundance. Because the soil beneath the colony trees is high in nutrients year-round (due to accumulated guano), soil-dwelling invertebrates are abundant, even outside the nesting season (Natusch et al. under review; Fig 3B). Those soil nutrients also promote the year-round growth of roots and seedlings, providing a rich resource for herbivores and insectivores—especially in the late dry season, when food resources in the surrounding landscape become scarce. Thus, pigs and birds of several species (especially brush turkeys) gather beneath the colony-trees primarily in the off-season. High abundances of predators during the nesting season also may threaten herbivores, and discourage them from using the areas under bird-colonies during the nesting season.

The most common pattern, though, is for species to be most common under bird-nesting trees (but not “random” trees) during the time when starlings are present. This pattern holds true for flying insects, nocturnally-active amphibians, reptiles, and birds. Most of these species obtain food directly via starlings’ activities. Especially after heavy rain, windy conditions cause nests (and entire tree-limbs) to fall to the ground. Predators (snakes, cane toads, dingoes, centipedes and birds of prey) feed on fallen starling eggs and nestlings, and invertebrates found beneath the trees (Fig 3). More generally, the temporal and spatial fluctuations in animal numbers reflect biotic interactions among the wildlife taxa involved, as well as between those species and the metallic starlings. For example, snakes and diurnal birds were more abundant beneath the largest colonies because of resources dropped by the starlings (e.g., seeds or fallen starling chicks). Other taxa simply utilize the secondary feeding opportunities created by the starling colonies (e.g., roots or invertebrates that grow or are attracted by the large quantities of bird guano)

Why are faunal concentrations so high beneath these trees compared to the rest of our study area? One plausible explanation is that the position of colony trees near the rainforest edge allows utilization of the resource by taxa inhabiting rainforest and woodland habitats. However, most of the taxa utilizing the trees are habitat generalists, and the concentrations observed are too great to be explained by subtle differences in habitat-specific faunal abundance. Furthermore, we did not survey all trees potentially suitable for starlings (because we cannot assume we know what attributes the birds require) and thus colony site location may simply be an artifact of tree distribution rather than because starlings actively select ecotonal areas for their colony sites

A more likely explanation is the ubiquitous nature of spatial and temporal variability in resource availability (and thus faunal abundance) [1]. Most spectacular examples of such heterogeneity generally involve escape from lethal abiotic extremes: such as communal overwintering dens of snakes to avoid freezing [21] or the restriction of some desert-dwelling anuran species to water-sources to avoid desiccation [22,5]. Such concentrations of fauna are driven by access to a scarce resource, critical for short-term survival. In contrast, aggregations driven by food availability tend to be less dramatic. Frugivorous birds and primates gather at fruiting trees [23], scavengers at drying pools [7], and sharks at whale carcasses [24]; but such aggregations tend to be short-term and involve relatively small numbers of animals. By contrast, the aggregations of wildlife under the bird-colony trees in Cape York last for months, are repeated each year, and involve massively higher densities of many species than can be found in the surrounding landscape.

In tropical Australia, the diverse range of animals utilising the starling-colony trees are undoubtedly attracted by the feeding opportunities available relative to other areas within the broader landscape. The low productivity and poor soils of our study area [25] increase this resource heterogeneity and thus, the faunal concentrations underneath the colony trees. Although this system is unique within Australia in terms of the magnitude of its influence, similar examples are widespread. For example, nesting aggregations of silver gulls (*Larus novaehollandiae*) on small islands support dense populations of predatory tiger snakes (*Notechis scutatus*: [26, 27]). Communally-breeding penguins (*Aptenodytes patagonicus*) and seals (*Arctocephalus gazella*) likewise support dense populations of marine predators [28, 29]. Marine birds and mammals often nest communally, because of limited terrestrial breeding sites within vast areas of oceanic feeding grounds [30]. For passerines, colonies of sociable weavers (*Philetairus socius*) in southern Africa and yellow-rumped caciques (*Cacicus cela*) in Peru are utilised by snakes and primates [10, 11]. Although these systems are superficially similar, the numbers and diversity of animals attracted to the colonies are relatively small compared to the starlings in our study. The most unique aspect of the starling system is its tiny point source (mean 140 m^2^ beneath colony trees). To our knowledge, this system represents one of the highest-biomass (up to 25 kg/m^2^ of pigs; based on a mean mass of 70 kg) and most diverse faunal aggregations in the world (42 different species; Table 1).

Starling colonies in northern Australia provide an ideal system for asking ecological and evolutionary questions. Future research could usefully explore how and why starlings choose their nesting sites, how animals locate colony trees, or how far they travel to reach them. The high concentrations of animals around starling colonies suggest that there are fitness benefits associated with utilization of this nutrient subsidy. Plausibly, starling colonies increase the number of animals that can be supported by the habitat, which also makes these trees disproportionately important for conservation [7]. High concentrations of invasive species (feral pigs and cane toads) utilizing the starling colonies is particularly noteworthy. Targeted control of feral animals at colony sites could improve conservation outcomes. To assist conservation endeavors, the spectacular nature of these animal concentrations and predator-prey interactions provide an opportunity to educate the public about the ecology of Australian tropical rainforests. Enthusing the general public about wildlife, and the dynamic nature of ecosystems, can play a critical role in building support for conservation initiatives. Our recent discovery of this phenomenon emphasizes the need for a continued role of naturalists in better understanding the Australian tropics.

## Supporting Information

S1 File Natusch et al. Camera trap data_PLOS ONE.xlsx. Caption: Raw camera trap data used in the analyses for this study.
(XLSX)

S2 File Natusch et al. Nocturnal surveys data_PLOS ONE.xlsx. (XLSX)
